# 6-Thioguanine and zebularine down-regulate DNMT1 and globally demethylate canine malignant lymphoid cells

**DOI:** 10.1186/s12917-014-0290-8

**Published:** 2014-12-06

**Authors:** Brian K Flesner, Senthil R Kumar, Jeffrey N Bryan

**Affiliations:** Department of Veterinary Medicine and Surgery, Comparative Oncology and Epigenetics Laboratory, College of Veterinary Medicine, University of Missouri-Columbia, 900 E. Campus Drive, Columbia, MO 65211 USA; Current address: School of Veterinary Medicine, Louisiana State University, Skip Bertman Drive, Baton Rouge, LA 70803 USA

**Keywords:** Lymphoma, Demethylation, Thioguanine, DNMT, Canine

## Abstract

**Background:**

The antimetabolite 6-thioguanine (6-TG) has been used to treat both human and canine lymphoid malignancies. 6-TG has been shown to be epigenetically active as a demethylating agent in a human lymphoma cell line, causing downregulation of DNA methyltransferase 1 (DNMT1) through ubiquitin-targeted degradation. Zebularine (Zeb), a similar cytidine analog, also has demethylating activity as well as oral bioavailability. The hypothesis of the present study was that 6-TG and Zeb would cause downregulation of DNMT1 and globally demethylate the genomic DNA of canine lymphoma cells. The secondary hypothesis was that these agents would cause a dose-dependent decrease in cell proliferation in canine lymphoma cells. Canine CLGL-90 malignant T cells and CLL 17–7 cells were incubated in modified RPMI media. They were treated with 6-TG, Zeb, or control media at biologically relevant concentrations.

**Results:**

Following treatment with each agent, DNMT1 protein and global DNA methylation were significantly decreased. A dose-dependent decrease in cell survival was also observed, with apoptosis being the primary mode of cell death in the CLGL-90 cell line.

**Conclusions:**

These results confirm the demethylating action of 6-TG and Zeb in canine cells which is similar to that shown in human cell lines. Confirmation of this mechanism supports the clinical application of these compounds as demethylating drugs in veterinary patients.

## Background

Lymphoma is the most commonly diagnosed hemo-lymphatic neoplasm of the dog [1]. Little progress has been made in remission and overall survival duration in the last few decades. Treatment involves cytotoxic chemotherapy with cyclophosphamide, doxorubicin, vincristine, prednisone (CHOP) protocols [2]. Despite an over 90% remission rate, almost all dogs relapse and eventually become resistant to cytotoxic chemotherapy drugs. Rescue protocols have been suboptimal, with median remission durations ranging between 40 and 60 days after initiation [3-7]. Recent work in human non-Hodgkin lymphoma (nHL), the group of diseases most similar to canine lymphoma, has identified multiple epigenetic mechanisms that could affect phenotype, aggressiveness, and response to therapy [8-10].

Demethylating agents have previously been used for human and canine hematopoietic malignancies [11-15], with approval of 5-aza-2'-deoxycytidine (decitabine) for human myelodysplastic syndrome and acute and chronic myelogenous leukemia [16]. Research in canine lymphoma has also identified epigenetic alterations, specifically methylation changes, associated with the neoplastic phenotype [17-19]. The protein family responsible for genomic methylation is a group of DNA methyltransferases (DNMTs); the specific maintenance methyltransferase protein in mammals is DNMT1 [20]. A recent human study showed that the drug 6-thioguanine (6-TG), a well-described antimetabolite agent, caused genomic demethylation by reduction of DNMT1 protein through the ubiquitin-proteasome pathway [21]. Because this drug has a known safety profile in dogs [11,12], it was selected for *in vitro* evaluation of its effect on global methylation on canine lymphoma cell lines. Zebularine (Zeb), a similar antimetabolite, also has been used *in vitro* and *in vivo* [22-24] and has recently been shown to be safe in the dog [C. Fulkerson, personal communication]. This led to similar interest in the compound for its demethylating mechanism of action. This drug was selected as a known demethylating comparator to the 6-TG. The hypotheses of these studies were that 6-TG and Zeb would reduce DNMT1 protein, leading to a decrease in global DNA methylation following exposure. The secondary hypothesis was that the drugs would decrease cell proliferation and induce apoptosis.

## Methods

### Cell culture

Two cell lines, a canine large T cell leukemia line CLGL-90 and a canine B cell line CLL 17–7 [25] were grown in custom RPMI media containing 1 mM sodium pyruvate, 10 mM HEPES, 2 mM L-glutamine, 0.4 mg/mL gentamicin, supplemented with 10% fetal bovine serum, and maintained at 37°C in a 5% CO2 air atmosphere. Cells were separated and placed into 6-well plates containing the same media. Initial experiments to monitor dose-relationship and cell survival between 6-TG (Sigma-Aldrich, St. Louis, MO) and Zeb (Zeb, Sigma-Aldrich, St. Louis, MO) were performed by dilution in PBS to yield final drug concentrations of 1.5, 3.0, and 6.0 μM [21] and 50, 100, and 200 μM respectively [22]. These biologically relevant concentrations are reported to be achievable *in vivo* [23,26]. Cells were incubated for 24 hours, mixed, and half of the cells and culture media were collected. Cells were centrifuged and the cell pellet was collected for DNMT1 and methylation analysis. The RPMI media was added to resuspend the remaining cells, and the previously dosed concentrations of 6-TG and Zeb were again added. The cells were again incubated for another 24 hours. A final collection was taken at 48 hours. Both 6-TG and Zeb were stored at −20°C after reconstitution for future experiments.

### DNMT1 western blot analysis

Protein was extracted from the same samples using MPER reagent (Thermo Fisher). Protein concentrations were measured (Nanodrop spectrophotometer) and samples were stored in −20°C. Equal amounts of protein (approximately 30 ug) extracted from the cell lines were separated by SDS–PAGE and blotted onto a PVDF membrane as described previously [21]. Membranes were blocked in 5% skimmed milk/Tris-buffered saline (TBS) for 1 h at room temperature. After washing with 1 × TBS the membrane was further incubated with DNMT-1 primary antibody (#5119; 700 μg/ml; 1:500) (Cell Signaling Technologies) and goat anti-rabbit secondary antibody (#7074;65 μg/ml;1:5000) (Cell Signaling Technologies). After evaluation via chemiluminescence (Kodak Image Station), membranes were stripped with antibody stripping buffer (Tris–HCl pH 6.8 with 2% SDS) containing mercaptoethanol. The blots were re-probed with antibody against Beta-actin (#4970; 21 μg/ml; 1:1000) (Cell Signaling Technologies), which served as a loading control. Relative intensity was evaluated using ImageJ (National Institutes of Health) to compare the intensity of each DNMT1 band to the loading Beta-actin band.

### Restriction landmark genomic scanning

DNA was extracted using a DNA extraction kit (DNeasy, Qiagen). DNA concentrations were measured (Nanodrop, Thermo-Scientific) and samples were immediately used or stored at −20°C. Global methylation was analyzed using restriction landmark genomic scanning (RLGS) as previously described [17]. RLGS produces varying lengths of DNA fragments dependent upon cytosine methylation status. Samples were incubated with either *Hpa*II or *Msp*I. *Hpa*II is unable to cleave DNA at its restriction site (CCGG) when cytosine is methylated, as it is a methylation-sensitive restriction endonuclease. However, its isochizomer *Msp*I is able to cleave the restriction site regardless of cytosine methylation status. As DNA becomes hypomethylated, *Hpa*II cleaves the strands more completely, resulting in smaller restriction fragments. This results in a change in intensity of the bands on DNA gel electrophoresis optical analysis. DNA (200 ng) was incubated with *Hpa*II (New England BioLabs) or *Msp*I (New England BioLabs) along with supplied NE Buffer and nuclease-free water for one hour at 37°C. After incubation, samples were electrophoresed on a 1% agarose gel at approximately 100 V/h. Comparison of the restriction enzymes’ (*Hpa*II vs. *Msp*I) banding patterns was used to determine alterations in the global methylation status of the DNA. Methylation score values (MSVs) were calculated for the samples [17]. The gel was photographed (UV Doc Station) and fragments in the 2.0-2.3 kb were analyzed using ImageJ. Measurements of the intensity of fragments were compared to background and MSVs were calculated using the following equation:

$$ \mathrm{M}\mathrm{S}\mathrm{V}=\frac{{\mathrm{Density}}_{\mathrm{HpaII}} - {\mathrm{Density}}_{\mathrm{Background}}}{{\mathrm{Density}}_{\mathrm{MspI}} - {\mathrm{Density}}_{\mathrm{Background}}} $$

### Cell survival analysis

CLGL-90 cells [25] were grown as described. Manual-count cell proliferation assays were performed. Cells were treated with increasing concentration of 6-TG (0, 1.5, 3.0, and 6.0 uM) and Zeb (with 0, 50, 100, and 200 uM) at 24 and 48 hours in replicates of three [21-24]. Cells were counted daily on a Bright Line hemocytometer (Hausser Scientific, Horsham, PA), with four grids being counted. An average of the four grids was calculated. Cells were diluted with Trypan Blue vital stain and the viable fraction was recorded at each time point. Dilution was appropriate to yield a minimum 100-cell count at each counting. Three replicates allow detection of a 25% difference with a power of 0.80 and alpha of 0.05. After evaluating the initial data derived from the increasing drug concentrations, the experiments were repeated at the highest concentrations of the drugs (6-TG at 6 uM and Zeb at 200 uM). Counts were made and compared at 24 and 48-hour time points.

### Apoptosis assay

Apoptosis assay was performed using Apo3/7 HTS assay kit according to the manufacturer’s (Cell Technology, Mountain View, CA) instructions. 15,000 cells were loaded per well in replicates of six for each treatment (control, 1.5 and 6 μM 6-TG and 50 and 200 μM Zeb) in 50 μl media, followed by addition of the apo HTS assay reagent containing quenched (z-DEVD)2-R110 dye substrate (50 μl). This was incubated for 1 h at 37°C. Active caspase 3/7 R110 cleaved the free dye, which excites at 488 nm, and the emission at 520 nm was read using a fluorescent plate reader.

### Statistical analysis

For cell survival analysis, treatment groups were compared by ANOVA (GraphPad Prism, GraphPad Software) with Holm-Sidak pair-wise comparison. One-way ANOVA was also used to compare methylation status. P-values less than or equal to 0.05 were considered significant.

### Ethics statement

The described experiments were conducted using existing, immortalized canine cell lines. No humans or vertebrate animals were used in the described studies.

## Results

### 6-TG and zebularine cause downregulation of DNMT1

**Figure 1:**
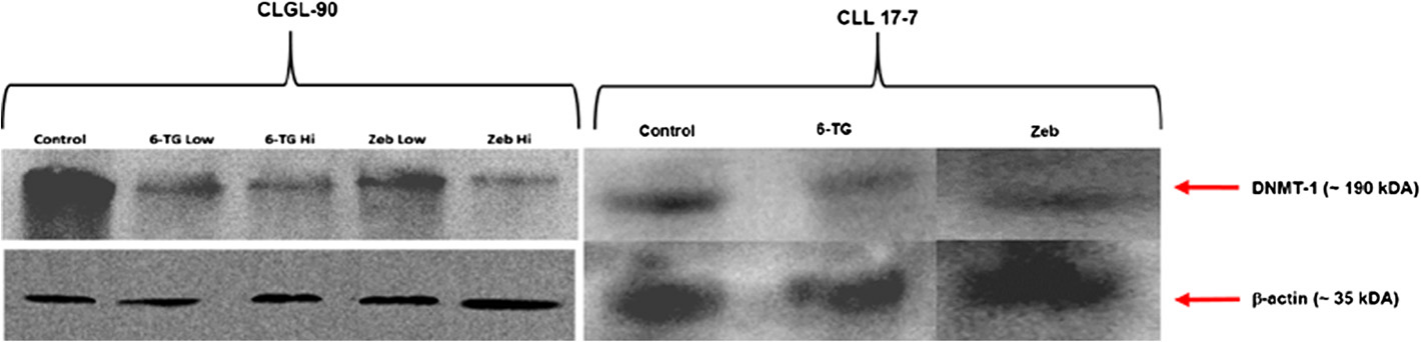
**Western blot of DNMT1 expression.** This image is a western blot of DNMT1 protein expression in CLGL 90 and CLL 17–7 cells treated with 1.5 μM or 6 μM 6-TG or 50 μM or 200 μM Zeb concentrations when compared to control. DNMT bands are evident at approximately 180,000 molecular weight. The treated groups had a reduction in intensity of DNMT1 expression as evident by bands in the 180 kDA region. Reduction in the expression of DNMT1 treated CLGL 90 cells (6-TG low, high and Zeb low, high groups) was 0.81, 0.76, 0.89, and 0.67 when compared to untreated control, respectively. Reduction in the expression of DNMT1 treated CLL 17–7 cells (6-TG and Zeb high dose) is 0.27 and 0.40, respectively. Loading control is Beta-actin.

To determine whether DNMT1 expression is reduced by 6-TG and Zeb treatment of canine cells, western blot analysis was performed. DNMT1 was reduced when treated with 6-TG and Zeb in both cell lines (Figure 1). The basal protein levels of DNMT1 were lower for the CLL 17–7 cell line than for the CLGL-90 cell line.

### 6-thioguanine and zebularine globally demethylate malignant lymphoid cells

**Figure 2:**
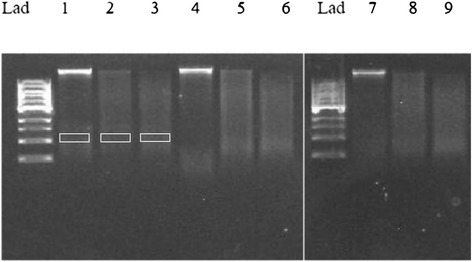
**DNA gel electrophoresis showing endonuclease activity in restriction landmark genomic scanning.** Lanes: Lad = ladder, 1 = Control, 2 = Control + Hpa, 3 = Control + Msp, 4 = 6TG, 5 = 6TG + Hpa, 6 = Hpa + Msp, 7 = Zeb, 8 = Zeb + Hpa, 9 = Zeb + Msp. 1 kb pair DNA ladders were loaded into lane 1, followed by control, *Hpa*II, and *Msp*I incubated extracted DNA from the lymphoma cell line for low and high doses of 6-TG and Zeb respectively. The MSV equation $\left(\mathrm{M}\mathrm{S}\mathrm{V}=\frac{\mathrm{Densit}{\mathrm{y}}_{\mathrm{HpaII}}\hbox{-}\ \mathrm{Densit}{\mathrm{y}}_{\mathrm{Background}}}{\mathrm{Densit}{\mathrm{y}}_{\mathrm{MspI}}\hbox{-}\ \mathrm{Densit}{\mathrm{y}}_{\mathrm{Background}}}\right)$ was used for each treatment subset. Densities were calculated at the 2.0-2.3 kb pair region. Boxes shown in the first three lanes demonstrate the region of analysis.

**Figure 3:**
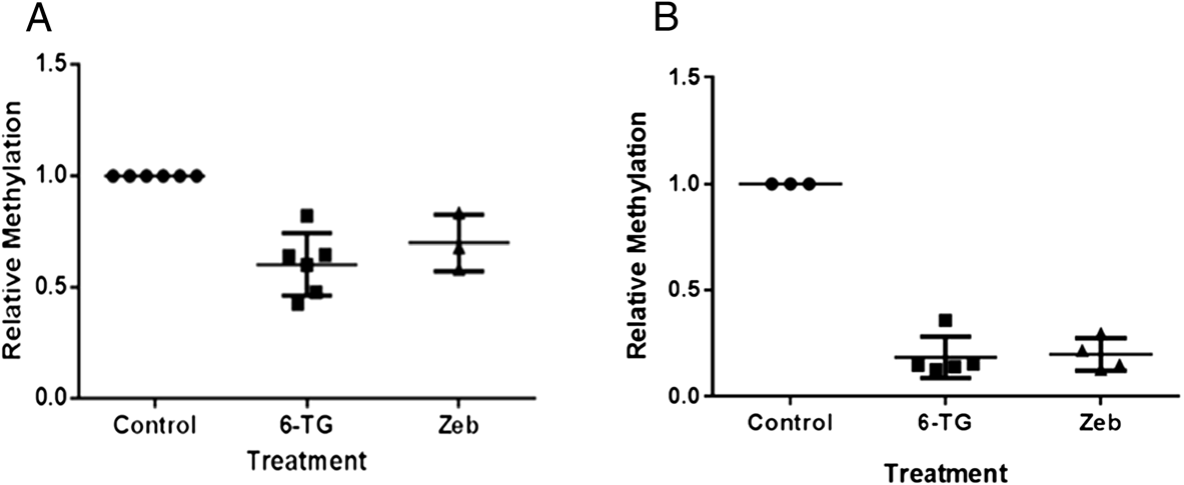
**A and B. Relative global DNA methylation with and without treatment by 6-TG or zebularine.** RLGS global methylation results after treatment with 6-TG and Zeb. 6-TG and Zeb had a statistically significant (P < 0.001) reduction in relative methylation when compared to control for each cell line. All results were normalized to control values of 1 for purposes of comparison. CLGL-90 is presented in panel A and CLL 17–7 in panel B. The central bar represents the mean of the data and error bars represent the standard deviation.

Global methylation was analyzed using RLGS, which produces varying lengths of DNA strands dependent upon cytosine methylation status. Samples were incubated with *Hpa*II (methylation-restricted) or *Msp*I (methylation unrestricted). Analysis of the resulting electrophoresis gels demonstrated significant decreases (P < 0.001) in global methylation following treatment with 6-TG and Zeb (Figure 2) in both cell lines. This effect was comparatively greater in the CLL 17–7 cell line than the CLGL-90 cell line (Figure 3A and B).

### 6-thioguanine and zebularine cause a dose-dependent reduction in cell survival

**Figure 4:**
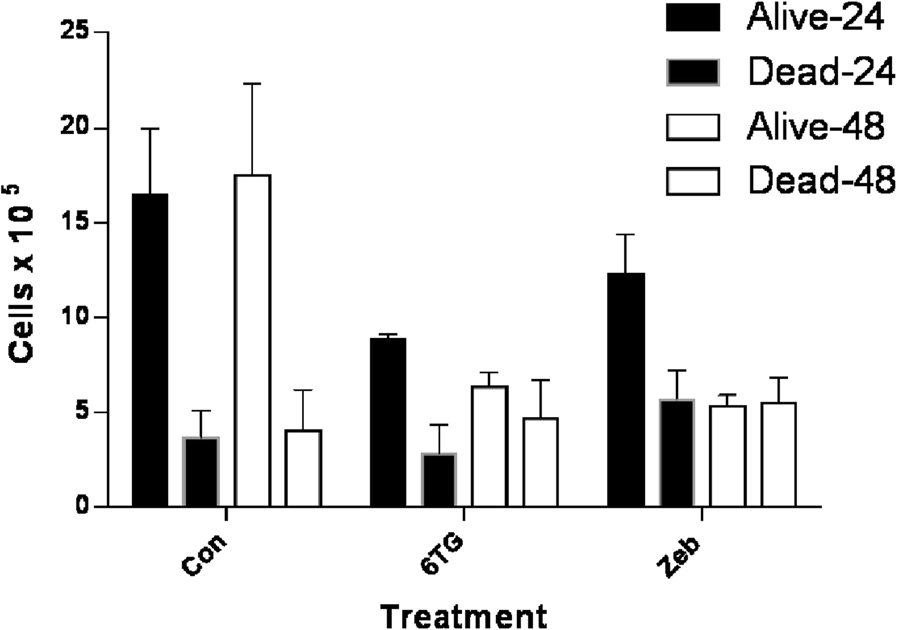
**CLGL-90 cell survival at 24 and 48-hour following treatment high dose 6-TG and zebularine compared to untreated controls.** 6-TG and Zeb were applied at 6 uM and 200 uM respectively. The number of alive and dead cells was significantly different for treated groups zebularine (p = 0.026) compared to controls, but not for 6-TG (p = 0.092). The error bars represent the standard deviation.

**Figure 5:**
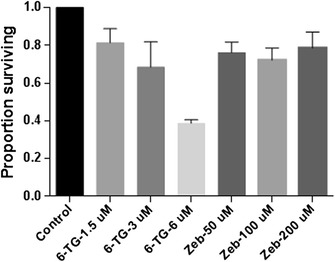
**Proportion of cells surviving following 48 hours of exposure to 6-G or Zeb normalized to controls.** Proportion of viable cells decreased significantly compared to control at 48 hours (P < 0.001). When comparisons were made inside groups, low vs. high treated cells showed a dose-dependent response to 6-TG (P = 0.013). The error bars represent the standard deviation.

CLGL-90 cells were plated at 2 × 10^5^ cells per well in 6 well plates. They were treated with either 0 (control), 1.5, 3.0, 6.0 μM 6-TG or 50, 100, 200 μM Zeb at time-points 0 and 24 hours. Cells were counted at 24 and 48 hours. There was a decrease in cell viability compared to control following exposure to Zeb at the highest concentration (P = 0.026) (Figure 4). 6-TG decreased the surviving fraction in a concentration dependent manner (P = 0.013) (Figure 5).

### Apoptosis

**Figure 6:**
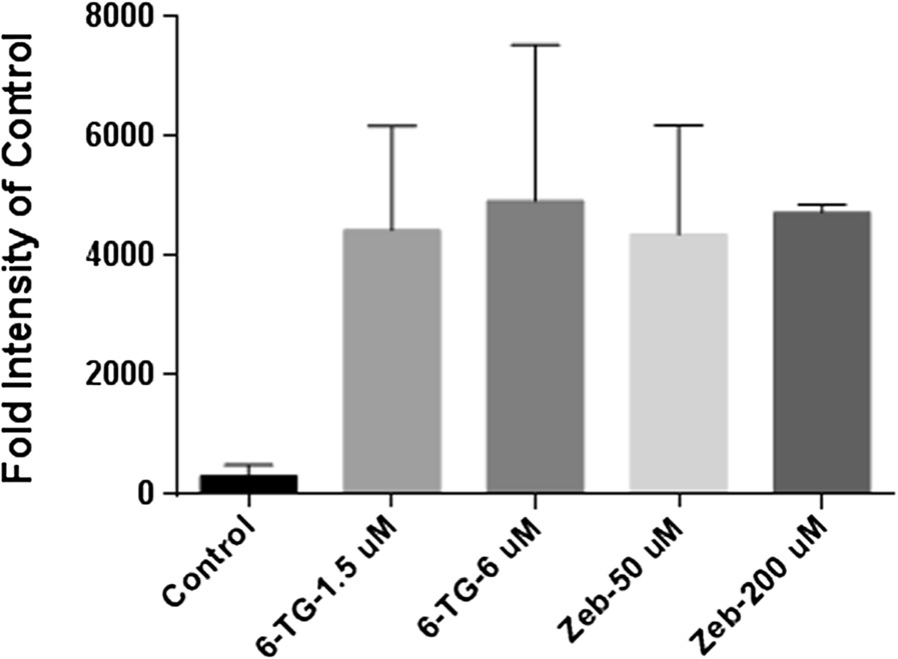
**Fluorescent intensity of activated Caspase 3/7 assay following treatment with 6-TG or Zeb.** Shown is the absolute intensity of fluorescence of 15,000 cells evaluated following no treatment (control) or low or high dose of 6-TG or Zeb. All treated groups are significantly different from control (P = 0.0011), but there is no difference between concentrations. The error bars represent the standard deviation.

Compared to control samples, treatment with both 6-TG and Zeb resulted in significantly increased Caspase 3/7 activity (P = 0.0011) (Figure 6). This increase in apoptosis was not dose-dependent for either drug.

## Discussion

Aberrant DNA methylation represents a novel target in the management of lymphoid malignancies. Evidence exists for hypermethylated tumor suppressor genes [18,19] and global DNA demethylation [17] in canine lymphoma. However, to date, the mechanism of demethylation by antimetabolites has not been demonstrated in malignant canine lymphoid cells.

The anti-metabolite 6-TG was recently shown to have DNA demethylating properties in a human lymphoma cell line [21]. With exposure to 6-TG, DNMT1 is shifted toward ubiquination-mediated proteasomal degradation via alteration of the expression of histone lysine-specific demethylase. While this specific mechanism was not confirmed in our study, we demonstrated similar decrease in DNMT1 protein in canine cells. Using western blotting techniques, we showed that both 6-TG and Zeb decrease DNMT1 protein in canine malignant lymphoid cells. This is a critical finding to support clinical application of demethylation therapy to canine cancers, and the first report of such a mechanism. Basal levels of DNMT1 were lower in the CLL 17–7 cell line, but the demethylating drugs still resulted in decreased protein expression. With the lower basal levels of this DNA methyltransferase, yet marked demethylation following treatment, it is possible that another protein in this family is more important to maintenance of methylation in this cell line.

The identification of global demethylation relative to control cells confirms the biologic effect of the decrease in DNMT1. The selectivity of demethylation by these agents is unknown. Further work is necessary to identify the genes and gene classes affected by these drugs. It is unclear if there is drug selectivity for therapeutically advantageous gene (i.e. tumor suppressor genes) activation, which could lead to cell cycle arrest of tumor cells. It is also unclear whether proto-oncogenes could become activated, promoting cells to enter the cell cycle. A potential pit-fall of this treatment could be tumor promotion if previously silenced pro-survival, anti-apoptotic, or other “aggressive” genes are awakened.

Because 6-TG is a purine antimetabolite, with activity against lymphoproliferative diseases [27,28], we investigated its effect on cell survival in CLGL-90. Both 6-TG and Zeb have a dose-dependent and cumulative effect on cell viability. Apoptosis was the mechanism of cell death, similar to the proposed mechanism of apoptosis or necrosis in human acute lymphoblastic leukemia Molt-4 T-cell treated with 6-TG *in vitro* [21]. However, the agents could also impede cell proliferation or cause cell cycle changes associated with methylation disturbances. Zebularine has been shown to be safe in small and large animal studies [24] with a similar demethylating mechanism. *In vivo* effects of Zeb in dogs have yet to be published, although preliminary evaluation of *in vivo* canine safety has been reported (Christopher Fulkerson, personal communication).

Our group has begun to investigate the strategy of priming with demethylation therapy immediately prior to chemotherapy in the relapse-resistant setting. A similar approach has been investigated in a mouse model of human diffuse large B cell lymphoma, giving decitabine prior to doxorubicin and CHOP chemotherapy for synergistic effect [29]. In immunocompromised mice, decitabine suppressed xenograft resistant lymphoma growth. Further, *SMAD1*, in the family of transforming growth factor-beta, was shown to be hypermethylated and silenced in the resistant lymphoma cell lines. Treatment with decitabine caused reactivation of SMAD1, and re-sensitization to doxorubicin *in vitro*. This treatment (decitabine priming followed by standard chemotherapy) was then administered to human patients, and *SMAD1* demethylation was shown in lymph node biopsies. The phase I clinical trial was safely administered and showed encouraging complete responses [29].

While this research is encouraging for future canine lymphoma treatment, it does have its limitations. All work reported was *in vitro*. The cell lines selected are traditional canine lymphoma cell lines, but were recently re-characterized as lymphoid leukemia cell lines [25]. Our group is currently looking at methylation status in normal lymph nodes and in lymphoproliferative disorders in untreated dogs. We plan to compare this to dogs receiving standard cytotoxic chemotherapy, with or without the addition of demethylating agents. If global demethylation is shown in the clinical model, we will explore which specific genes, especially tumor suppressor genes, are affected by methylation. This work does raise the concern of activating oncogenes, and whether demethylating agents might actually confer a phenotypic advantage to aggressive lymphoma. Tomiyasu and others compared drug-sensitive canine lymphoma cell lines to drug-resistant lines. The *ABCB1* gene associated with multi-drug resistance was hypomethylated in the drug-resistant cell lines [30]. This finding suggests that further evaluation of the exact mechanism and effect of demethylating drugs in tumor-bearing dogs is warranted.

While our research investigates canine lymphoproliferative disorders, human clinical trials have shown significant clinical responses in patients with other, resistant myeloproliferative disorders (MPD) [16]. While these drugs have not been investigated prospectively in MPD in veterinary medicine, the safety of 5-azacitidine (Vidaza), a demethylating agent, has been demonstrated in dogs with naturally occurring urothelial carcinoma [31]. Partial responses or stable disease were seen in the majority of dogs. Myelosuppression was the primary side effect noted. We expect the investigation of mechanism of action and therapeutic utility of epigenetic modifiers in veterinary medicine to flourish, hopefully leading to increased efficacy and survival when used with current therapies.

## Conclusions

These results confirm the demethylating action of 6-TG and Zeb in canine cells, confirm a similar demethylating mechanism as that shown in human cell lines, and support the need for *in vivo* research to determine whether these compounds could improve response to therapy in dogs with resistant lymphoma.

## Abbreviations

6-TG: 6-thioguanine
DNMT1: DNA methyltransferase 1
CHOP: Cyclophosphamide, doxorubicin, vincristine, and prednisone multi-agent chemotherapy
nHL: Non-Hodgkin lymphoma
Zeb: Zebularine
MSV: Methylation score value
ABCB1: ATP-binding cassette subfamily B, member 1
MPD: Myeloproliferative disorders
